# Oral Administration of Penicillin or Streptomycin May Alter Serum Serotonin Level and Intestinal Motility via Different Mechanisms

**DOI:** 10.3389/fphys.2020.605982

**Published:** 2020-12-23

**Authors:** Cuihong Zhu, Huashan Gong, Ping Luo, Li Dong, Guohua Zhang, Xueyin Shi, Weifang Rong

**Affiliations:** ^1^Department of Anesthesiology, Xinhua Hospital, Shanghai Jiao Tong University School of Medicine, Shanghai, China; ^2^Department of Anatomy and Physiology, Shanghai Jiao Tong University School of Medicine, Shanghai, China

**Keywords:** intestinal microflora, enterochromaffin cells, hyperpolarization-activated cyclic nucleotide-gated channels 2, tryptophan hydroxylase 1, serotonin, antibiotics

## Abstract

**Background/Aims:**

Enterochromaffin cells (EC cells) constitute the largest population of enteroendocrine cells and release serotonin (5-HT) in response to mechanical and chemical cues of the gastrointestinal tract (GIT). How EC cells respond to altered microbiota such as due to antibiotic treatments remain poorly understood. We hypothesized that the pacemaker channel HCN2 might contribute to the regulation of EC cells functions and their responses to antibiotics-induced changes in intestinal flora.

**Methods:**

Mice were given either penicillin or streptomycin or both in drinking water for 10 consecutive days. The changes in the profile of short chain fatty acids (SCFAs) in the cecum following penicillin or streptomycin treatments were tested by GC-MS. Serum 5-HT content, whole intestinal transit time, fecal water content, cecum weight and expression of HCN2 and TPH1 in cecal mucosa were measured. Ivabradine (a HCN channels blocker) was used to explore the role of HCN2 in penicillin-induced changes in 5-HT availability and intestinal motility.

**Results:**

HCN2 immunofluorescence was detected on intestinal EC cells. Both penicillin and streptomycin caused significant reduction in total SCFAs in the cecum, with the penicillin-treated group showing greater reductions in butyrate, isobutyrate and isovalerate levels than the streptomycin group. The expression of HCN2 was increased in the mice treated with penicillin, whereas TPH1 expression was increased in the mice treated with streptomycin. Mice treated with antibiotics all had larger and heavier cecum, elevated serum 5-HT level and increased fecal water content. Besides, mice treated with penicillin had prolonged intestinal transit time. Intraperitoneal injection of Ivabradine attenuated the effect of penicillin on serum 5-HT level, cecum size and weight, intestinal motility, and fecal water content.

**Conclusion:**

Disruptions of the intestinal flora structure due to oral administration of penicillin may significantly increase serum 5-HT level and inhibit intestinal motility, at least partially through up-regulating the expression of HCN2. Oral administration of streptomycin may alter 5-HT availability by up-regulating TPH1 expression thus increasing synthesis of 5-HT. Alterations of intestinal flora composition due to exposure to different antibiotics may regulate 5-HT availability and intestinal motility through different mechanisms.

## Introduction

The human gastrointestinal tract (GIT) is inhabited with tens of trillions of microorganisms (Kau et al., 2011). The vast majority of intestinal bacteria, estimated to be as many as 3.8 × 10^13^, exist in the colon (Sebastian Domingo and Sanchez Sanchez, 2018). In addition, the cecum has been considered as a reservoir and a “safe house” for anaerobic bacteria to rapidly populate the colon (Sahami et al., 2016; Brown et al., 2018). Increasing evidence suggest that the intestinal flora exert major influences on the host homeostasis. Dysbiosis of the intestinal flora may be closely related to the occurrence and development of extra-GIT as well as GI diseases, such as inflammatory bowel diseases, colorectal cancers, and the autism spectrum disorders (Joossens et al., 2011; Vuong and Hsiao, 2017; Wong and Yu, 2019).

The interaction between the intestinal flora and the host is immensely complex. The enteroendocrine cells, which constitute approximately 1% of the total intestinal epithelial cells, are strategically located to sense the changes of intestinal microenvironment, including variations of the intestinal flora and their metabolites (Martin et al., 2017; Lund et al., 2018). Amongst the numerous types of enteroendocrine cells, the serotonin-releasing enterochromaffin (EC) cells are most numerous and are scattered diffusively throughout the entire gastrointestinal tract with uneven distribution. It was reported that the great majority of EC cells are scattered in the small intestine and colon in human beings; in rats, however, EC cells are mainly located in cecum and proximal colon (Qin et al., 2019). The serum 5-HT level of germ-free mice was only 40% of that of conventional mice, and transplantation of normal intestinal flora into germ-free mice restored the serum 5-HT level similarly to that of conventional mice (Reigstad et al., 2015). Moreover, treatment of primary cultured EC cells with high concentration of short chain fatty acids (SCFAs) led to increased expression of tryptophan hydroxylase 1 (TPH1), the rate-limiting enzyme for 5-HT synthesis in EC cells (Lyte, 2013). Recent data also suggest that 5-HT released from EC cells to the lumen may modulate bacterial colonization in the gut (Fung et al., 2019). These data suggest that EC cells may be an important interface between the intestinal flora and the host.

Antibiotics are the most important treatments for infectious diseases. Dysbiosis of the intestinal flora associated with antibiotic treatment may potentially alter the synthesis and the release of 5-HT from EC cells thereby impacting on host homeostasis, particularly the GI functions. Indeed, antibiotic exposure reportedly may either increase or decrease 5-HT availability, depending on types of antibiotics used and animal species studied (Ge et al., 2017; Hasani et al., 2019). A better understanding of how antibiotic exposure may alter synthesis and release of 5-HT from EC cells may provide important insights on the prevention and management of adverse effects of antibiotics.

Hyperpolarization-activated cyclic AMP-gated cation channel-2 (HCN2) is a unique ion channel, initially identified in cardiac pacemaker cells. When the membrane potential repolarizes to near the resting potential level or the concentration of intracellular cAMP was increased, the HCN2 channel can be activated, resulting in an inward current (Ih) promoting excitation of excitable cells (Emery et al., 2011). Previous studies have demonstrated functional HCN2 channel in several types of endocrine cells, such as pituitary lactotrophs (Calejo et al., 2014), insulin-secreting islet cells (Zhang et al., 2009), and the GLP-1-secreting intestinal L-cells (Stanley et al., 2004; Reimann et al., 2005). It is well-known that cholera toxin stimulates intestinal electrolyte secretion by elevating cAMP (Hudson et al., 1975; Epple et al., 1997). Some products of commensal bacteria, such as SCFAs, may also alter intracellular cAMP through Gs or Gi/o-coupled receptor (Brown et al., 2003; Le Poul et al., 2003). We therefore speculate that HCN2 might be expressed on EC cells and may contribute to the regulation of 5-HT availability following antibiotics exposure.

To test the above hypothesis, we determined HCN2 expression on EC cells through immunofluorescence and examined the effects of oral administration of penicillin and streptomycin, either alone or in combination, on serum 5-HT level, intestinal motility as well as HCN2 and TPH1 expression. Penicillin is a β-lactam antibiotic, which inhibits replication of Gram^+^ bacteria by interfering with cell wall formation, whilst streptomycin is an aminoglycoside antibiotic, which kills Gram^–^ bacteria through inhibition of protein synthesis. Exposure of mice to penicillin was found to cause an increase in serum 5-HT level and inhibition of intestinal motility, which was associated with upregulation of HCN2 expression. Exposure to streptomycin also led to elevation of serum 5-HT, which was associated with upregulation of TPH1. Our data indicate that antibiotics exposure may regulate peripheral 5-HT availability and intestinal motility through different mechanisms, depending on the antibacterial spectrum.

## Materials and Methods

### Mice and Treatments With Antibiotics

Specific pathogen free (SPF) C57BL/6 mice (7–8 weeks old) were purchased from Shanghai Lingchang Biotechnology Co., Ltd., and housed in a temperature-controlled (23–25°C) room within the animal facility of Shanghai Jiao Tong University School of Medicine. All procedures were conducted within the facility and the mice were allowed free access to water and standard laboratory rodent chow, with a 12 h light and 12 h dark cycle. All animal care and study protocols were performed in accordance with the Guiding Principles in the Care and Use of Animals and the Animal Management Rule of the Ministry of Public Health, China (documentation 545, 2001) and approved by the Ethnic Committee for Experimental Use of Animals of Shanghai Jiao Tong University School of Medicine (document #SYXK-2013-0050).

Mice were treated with antibiotics in drinking water for 10 days to disrupt the intestinal flora compositions. Sterile water containing penicillin G (2 mg/mL, MCE, United States), streptomycin (4 mg/mL, Yuanye Biology, China) or penicilin + streptomycin (2 mg/mL and 4 mg/mL) was available *ad libitum* during the treatments (*n* = 5–7 mice for each group). The dose of antibiotics used was in accordance with previous studies (Ali et al., 2011; Sato et al., 2016). Weight of mice were measured daily during the period of antibiotic treatment. Treatment with the antibiotics did not significantly affect weight gain (Supplementary Figure 1). To evaluate the role of HCN2 channels in the changes of serum 5-HT and intestinal motility in penicillin-treated mice, Ivabradine (Iva), a blocker of HCN channels in clinical use for the treatment of arrhythmia and heart failure, was given by intraperitoneal injection (20 mg/kg, Biorbyt, England) daily during penicillin treatment. Another group of penicillin-treated mice was given intraperitoneal injection of saline as control (*n* = 5–10 mice for each group).

### Whole Intestinal Transit

On day 11, mice were orally gavaged with 6% carmine dye (Sigma, United States) in 200 μL 0.5% methyl cellulose (Sigma, United States) (Golubeva et al., 2017). The fecal pellets of mice were monitored at 15 min intervals and the time for excretion of the first red stool was recorded as an index of the whole intestinal transit.

### Fecal Water Content

Fecal particles were collected within 6 h and then the fecal samples were weighed before and after desiccation at 55°C for 22 h. The weight difference was recorded as fecal water content (Nezami et al., 2014).

### Serum and Tissue Samples

After intestinal motility test, mice were euthanized by an overdose of sodium pentobarbital. We focused on cecum tissue since we noted significant changes in the cecum size following antibiotics treatments. The total weight and net weight of cecum were measured. Serum samples were collected and stored at −80°C for the detection of 5-HT content. The cecal content was collected and stored at −80°C for detection of short chain fatty acids. The cecal segments were excised and cut longitudinally, and then washed in cold saline. The mucosa of the cecum was scraped off and cryopreserved for western blot detection of HCN2 and TPH1 expression levels.

### Western Blot

The cecal epithelium samples were homogenized in the lysis buffer and then centrifuged (10000 *g*) for 30 min at 4°C. Tissue lysis buffer contained 20 mmol/L Tris–HCl (pH 8.0), 1 mmol/L PMSF, 150 mmol/L NaCl, 1% NP-40, 1 mmol/L EDTA, protease inhibitor cocktail (Sigma, St. Louis, MO, United States) and phosphatase inhibitor cocktail (Thermo, Indianapolis, IN, United States). Total protein concentration in the supernatants was determined by BCA assay (Pierce, Rockford, IL, United States). Twenty-two microgram protein of each sample was loaded on 4–8% Tris-glycine ready gel (Bio-Rad, Hercules, CA, United States), and the size-separated proteins were transferred from the gel to a PVDF membrane (Bio-Rad, Hercules, CA, United States). After blockade with 5% fat-free milk in Tris-buffered saline (TBS) containing 0.1% Tween-20 for 2 h at room temperature, the membrane was incubated overnight at 4°C with rabbit anti-HCN2 (1:400, Alomone, Israel) and rabbit anti-TPH1 (1:500, Millipore, United States). The membrane was washed with PBS buffer and then incubated with HRP-conjugated anti-rabbit secondary antibody (1:3000, Bio-Rad) for 1 h at room temperature to identify the protein bands of HCN2 and TPH1. For detection of β-actin, the membrane was then incubated with 3–4 mL striping buffer (Thermo Fisher Scientific, United States) at 37°C for 1 h, washed with TBS buffer for three times and subsequently incubated with mouse anti-β-actin (1:3000, Abcam) overnight and HRP-conjugated anti-mouse secondary antibody (1:3000, Bio-Rad) for 1 h. Protein bands were detected with enhanced chemiluminescence (Thermo, Indianapolis, IN, United States), and the digital imaging was captured with Image Quant LAS 4000 mini (GE Healthcare, Life Science). The density of target protein bands was measured with NIH ImageJ software and normalized with the density of β-actin.

### Immunofluorescence

To detect HCN2 immunofluorescence in murine intestine, mice were euthanized with an overdose of pentobarbital and were prefixed through transcardiac perfusion with saline followed by 4% paraformaldehyde (PFA). Intestinal tissues were removed and post-fixed with 4% PFA overnight at 4°C. The fixed tissues were transferred to 30% sucrose for dehydration until the tissue had sank to the bottom of centrifuge tube. Then the specimens were embedded with OCT. For immunofluorescence staining, 10 μm-thick frozen sections were cut. Tissue sections were washed four times with PBS and blocked with 3% BSA and 10% normal donkey serum in PBS contained 1% TritonX-100 for 1 h at room temperature. Then tissue sections were incubated with rabbit anti-HCN2 (1:1000, Alomone, Israel) or goat anti-5-HT (1:2000, Abcam, Cambridge, MA, United States) at 4°C for 48 h. After four rinses with PBS, the sections were incubated with donkey anti-rabbit Alexa Fluor 488 secondary antibody (1:1000, Invitrogen, Eugene, OR, United States) or donkey anti-goat Alexa Fluor 568 secondary antibody (1:1000, Invitrogen, Eugene, OR, United States) for 2 h at room temperature. After washing with PBS, the sections were mounted onto glass slides with mounting medium, then observed and photographed using fluorescent microscope (Leica DM 2500, Germany).

### Determination of 5-HT Concentration by ELISA

Serum 5-HT concentrations were measured using the Serotonin ELISA Kit (LDN, Germany) according to the manufacturer’s instructions. The concentration of 5-HT in each sample was extrapolated from the standard curve.

### Quantification of Short Chain Fatty Acids

The cecum content of mice from different groups was sonicated in 0.005 M aqueous NaOH (at 4°C) and centrifuged. Then supernatant was collected for detection of short chain fatty acids (SCFAs) by gas chromatography-mass spectrum (GC-MS) using an Agilent 7890B gas chromatography system coupled to an Agilent 5977A mass spectrometric detector (MSD, Agilent Technologies, Santa Clara, CA, United States).

### Data Analysis

All values are presented as mean ± SEM. Statistical analysis was performed by GraphPad Prism 7 (La Jolla, CA, United States). Two-way ANOVA was used to compare differences among multiple groups. Student’s *t*-test was used to compare the difference between two groups. Differences were considered statistically significant when the *P* value was less than 0.05.

## Results

### Murine Intestinal Enterochromaffin Cells Express HCN2 Channels

**FIGURE 1:**
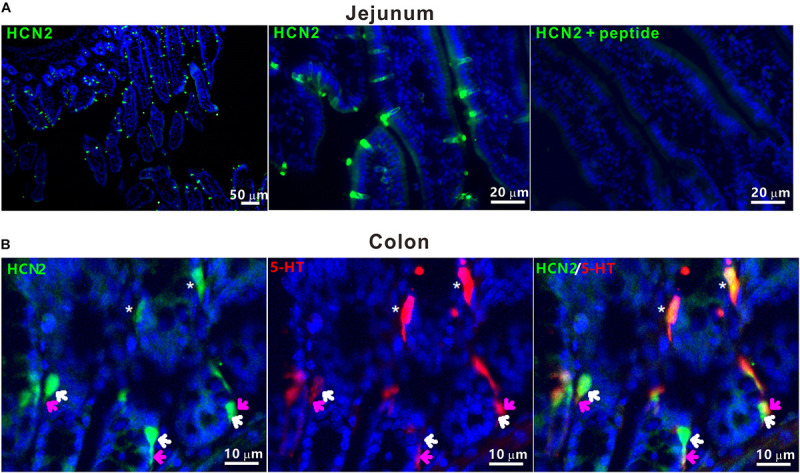
HCN2 channels were expressed on murine enterochromaffin cells. **(A)** Representative microphotographs of HCN2 immunofluorescence (green) in mouse jejunum. Specificity of antibody for HCN2 was confirmed by absorption test. **(B)** Representative microphotograph of HCN2 (green) and 5-HT (red) immunofluorescence in mouse colon. The asterisks indicate co-labeling of HCN2 and 5-HT immunoreactivity in EC cells. White (HCN2) and pink (5-HT) arrows indicate HCN2 and 5-HT immunoreactivity in different enteroendocrine cells in close proximity. Images are representative of at least three experiments.

Previous studies have shown functional HCN2 channel in GLP-1-secreting intestinal L-cells (Reimann et al., 2005). To determine whether HCN2 channel is also expressed in enterochromaffin (EC) cells, we conducted immunofluorescent staining of the murine intestinal tissues. HCN2 immunoreactive cells were detected in epithelium of the duodenum, the jejunum, the ileum and the colon. Figure 1 shows typical HCN2 immunofluorescence in murine jejunum and colon. Double immunofluorescence assay confirmed colocalization of HCN2 immunoreactivity with 5-HT in EC cells. Besides, there also exist some HCN2 positive (but 5-HT negative) cells in close proximity to 5-HT positive cells. This is consistent with previous findings that other enteroendocrine cells (e.g., GLP-1-secreting L cells) may also express HCN2. These results suggest that the pacemaker channel HCN2 may play a role in regulation of 5-HT release from EC cells.

### Co-administration of Penicillin and Streptomycin Results in Up-Regulated HCN2 Expression, Elevated Serum 5-HT Level and Enlargement of the Cecum

**FIGURE 2:**
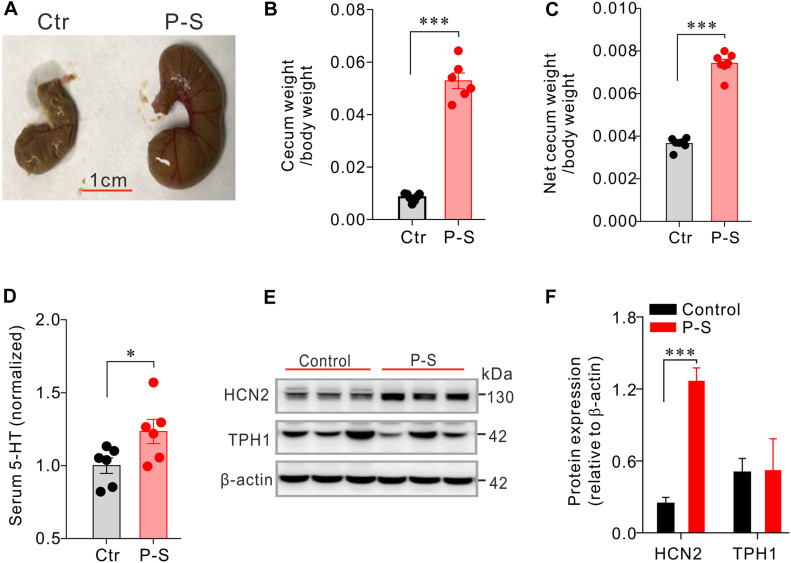
Co-administration of penicilin-streptomycin (P-S) caused significant changes in serum 5-HT, cecum size and weight and HCN2 expression in cecal epithelium. **(A)** Photos of the typical cecum of control and P-S-treated mice. **(B,C)** Comparisons of the wet (with cecum content) and dry (net) cecum weight (normalized to the body weight) between P-S-treated and control mice. **(D)** Comparisons of the serum 5-HT content between P-S-treated and control mice. Data were normalized to serum 5-HT content in control mice. **(E)** Western blot detection of HCN2, TPH1, and β-actin in cecal mucosa of the control mice and the mice treated with P-S. **(F)** Quantification of relative HCN2 and TPH1 protein in the cecal mucosa. ^∗^*P* < 0.05 and ^∗∗∗^*P* < 0.001 vs. control. *n* = 6 mice for each group.

To test whether antibiotics-induced disruption of the intestinal flora may alter serum 5-HT level, a group of mice (*n* = 6) were co-administered with penicillin and streptomycin (P-S) in drinking water for 10 consecutive days. Control mice (*n* = 6) were given normal drinking water. It was quite apparent that the P-S group had larger cecum. Accordingly, P-S-treated mice had greater wet cecum weight/body weight (0.0585 ± 0.00057 vs. 0.053 ± 0.0030, *P* < 0.001) and net cecum weight/body weight (0.0037 ± 9.8e-005 vs. 0.0074 ± 0.00020, *P* < 0.001) (Figures 2A–C) compared with the control mice. The serum 5-HT concentration of mice treated with P-S was significantly higher than that of the control mice (*P* < 0.05, Figure 2D). Additionally, it was found that HCN2 expression in the cecal epithelium was significantly higher in P-S-treated mice than the control mice (0.24 ± 0.053 vs. 1.26 ± 0.11, *P* < 0.001) (Figures 2E,F). However, TPH1 expression was not significantly different between the two groups. These data show that intestinal dysbiosis resulting from co-administration of penicillin and streptomycin may impact EC cells and alter the peripheral 5-HT level.

### Penicillin and Streptomycin Differentially Alter the Profile of SCFAs in the Cecum

**FIGURE 3:**
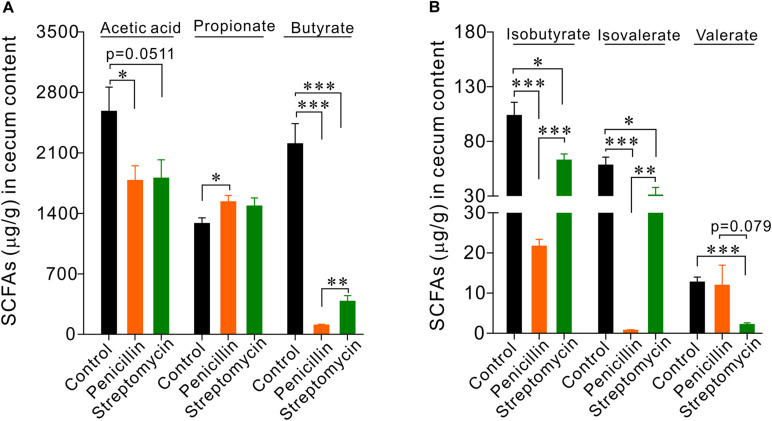
GC-MS analysis of short chain fatty acids (SCFAs) in cecum contents of mice. **(A)** Statistical analysis of acetate, propionate, and butyrate. **(B)** Statistical analysis of isobutyrate, isovalerate, and valerate. ^∗^*P* < 0.05, ^∗∗^*P* < 0.01, and ^∗∗∗^*P* < 0.001. *n* = 5 for each group.

Penicillin and streptomycin have different antibacterial spectrum. Oral administration of penicillin or streptomycin alone likely may result in differences in intestinal flora compositions, thereby may impact EC cells and intestinal function differently. To test this possibility, we first observed the effects of oral administration of penicillin or streptomycin on the level of short-chain fatty acids (SCFAs) in the cecum. Thus, two groups of mice (*n* = 5 each) were given 10 days of penicillin and streptomycin in drinking water, respectively. A control group (*n* = 5) were given normal drinking water. Following the treatment, the level of SCFAs in the cecum content of the mice was analyzed by GC-MS. Penicillin or streptomycin treatment both led to significant reductions of acetic acid, butyrate, isobutyrate, and isovalerate levels but slight increases of propionate in the cecum, compared with the control group (Figure 3). The penicillin group showed greater decreases in butyrate, isobutyrate, and isovalerate level than the streptomycin group. The streptomycin group but not the penicillin group showed significant reduction in valerate level. The differences in the profile of cecum SCFAs between the penicillin and the streptomycin groups support the notion that the two antibiotics may differentially alter the intestinal flora composition in mice.

### Mice Treated With Penicillin Alone Had Larger Cecum, Elevated Serum 5-HT and Up-Regulation of HCN2 Expression

**FIGURE 4:**
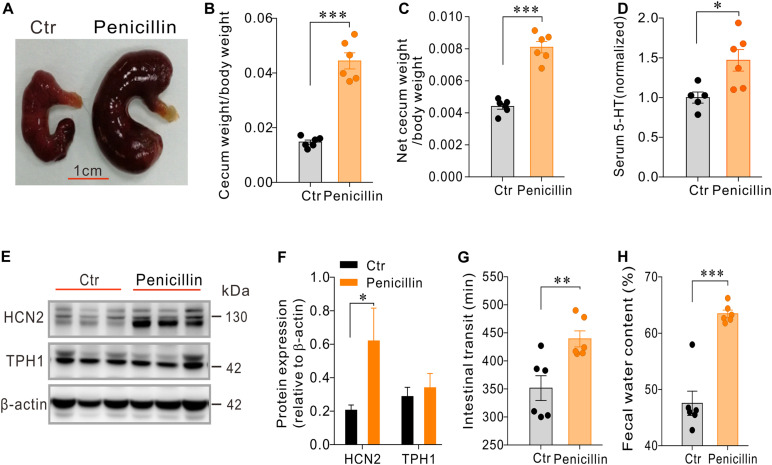
Effects of penicillin treatment on serum 5-HT level, cecum size and weight, intestinal motility and expression of HCN2 and TPH1 in cecal epithelium. **(A–C)** Comparison of cecum size and weight between the penicillin-treated and control mice. **(D)** Comparison of the serum 5-HT content between the penicillin-treated and the control mice. Data were normalized to serum 5-HT content in control mice. **(E)** Western blot detection of HCN2, TPH1, and β-actin in the cecal mucosa of the control mice and the mice treated with penicillin. **(F)** Quantification of relative HCN2 and TPH1 protein in the cecal mucosa. **(G)** Intestinal transit time of the mice detected by carmine dye test. **(H)** Fecal water content of the mice. ^∗^*P* < 0.05, ^∗∗^*P* < 0.01, and ^∗∗∗^*P* < 0.001. *n* = 6 mice for each group.

In another cohort of mice, we found that mice treated with penicillin alone (*n* = 6) had larger and heavier cecum compared with the control mice (*n* = 6) (Figures 4A–C). Serum level of 5-HT was also significantly increased in penicillin-treated mice than the control mice (*P* < 0.05, Figure 4D). Western blot showed that, compared with the control group, penicillin-treated mice had significant up-regulation of HCN2 expression in cecal mucosa (*P* < 0.05, Figures 4E,F). However, the expression of TPH1 protein was not significantly different between the penicillin-treated mice and the control mice. Immunofluorescent staining of the colonic tissues showed that the number of 5-HT^+^ EC cells were not significantly different between the two groups (Supplementary Figure 2), suggesting that the increase in serum 5-HT levels in the penicillin-treated mice was independent of altered EC cell numbers.

It is well known that 5-HT secreted by EC cells may influence intestinal motility and secretion (Kojima et al., 2017; Bhattarai et al., 2018; Stakenborg et al., 2019). We therefore examined how penicillin-treatment may affect the whole intestinal transit and fecal water content. Carmine dye test showed that intestinal transit time of penicillin-treated mice was significantly prolonged than that of the control mice (351.5 ± 22.07 vs. 439.3 ± 14.3 min, *P* < 0.01) (Figure 4G). Moreover, we found that fecal water content of the mice treated with penicillin was also significantly increased than that of the control mice (47.54 ± 2.16 vs. 63.45 ± 0.66%, *P* < 0.001) (Figure 4H). The slowed intestinal transit and increased fecal water content tend to suggest that penicillin exposure may inhibit intestinal motility but increase epithelial fluid secretion.

### Mice Treated With Streptomycin Alone Had Larger Cecum, Elevated Serum 5-HT and Up-Regulation of TPH1 Expression

**FIGURE 5:**
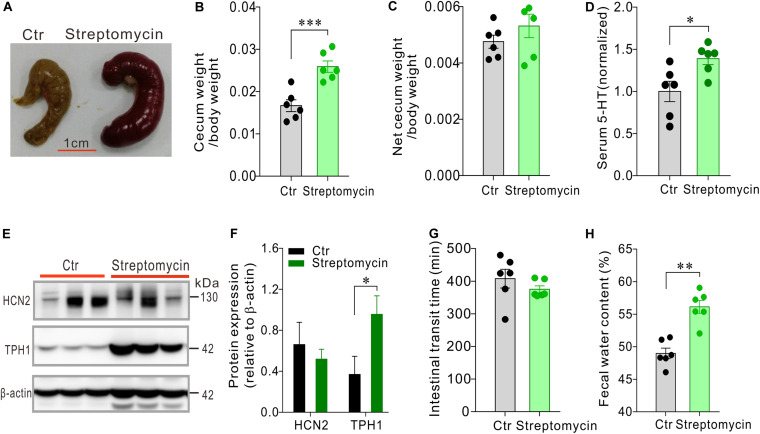
Effects of streptomycin treatment on serum 5-HT level, cecum size and weight, intestinal motility and expression of HCN2 and TPH1 in the cecal epithelium. **(A–C)** Comparison of cecum size and weight between the streptomycin-treated and control mice. **(D)** Comparison of the serum 5-HT content between the streptomycin-treated and the control mice. Data were normalized to serum 5-HT content in control mice. **(E)** Western blot detection of HCN2, TPH1, and β-actin in the cecal epithelium of the control mice and the mice treated with streptomycin. **(F)** Quantification of relative HCN2 and TPH1 protein in the cecal mucosa. **(G)** Intestinal transit time of the mice detected by carmine dye test. **(H)** Fecal water content of the mice. ^∗^*P* < 0.05, ^∗∗^*P* < 0.01, and ^∗∗∗^*P* < 0.001. *n* = 6 mice for each group.

We then asked how streptomycin, with a different antibacterial spectrum than that of penicillin, would alter serum 5-HT level and intestinal motility. Streptomycin-treated mice (*n* = 6) also had larger and heavier cecum than the control mice (*n* = 6) (Figures 5A–C), albeit cecum enlargement appeared to be less pronounced than penicillin-treated mice. The serum 5-HT level was significantly increased in the streptomycin-treated mice compared with the control group (*P* < 0.05, Figure 5D). Western blot showed that expression of HCN2 in cecal epithelium of streptomycin-treated mice was not significantly different from that of the control mice. However, expression of TPH1 was significantly up-regulated in streptomycin group compared with the control mice (*P* < 0.05, Figures 5E,F). Streptomycin treatment had no significant effect on intestinal transit time, but led to a significant increase in fecal water content (*P* < 0.01, Figures 5G,H). The above results suggested that streptomycin treatment may cause changes in 5-HT availability and intestinal function through a mechanism different from penicillin treatment.

### HCN Channel Blocker Ivabradine (Iva) May Attenuate the Effects of Penicillin Treatment on Serum 5-HT and Intestinal Motility

**FIGURE 6:**
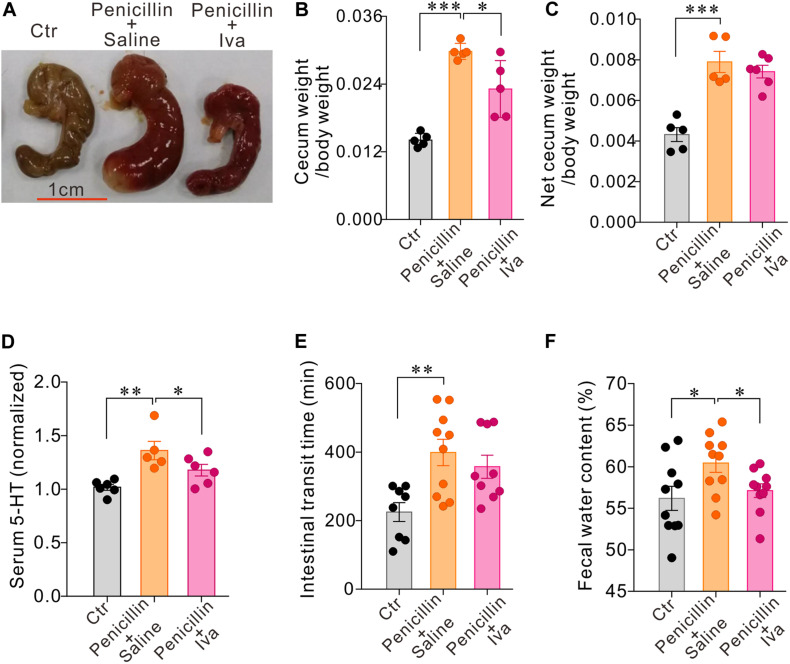
HCN channels blocker Ivabradine (Iva) attenuated the effect of penicillin on serum 5-HT level and intestinal motility. **(A–C)** Comparisons of the cecum size and weight amongst three groups of mice. **(D)** Serum 5-HT levels in three groups of mice. Data were normalized to serum 5-HT content in control mice. **(E)** Intestinal transit time detected by carmine dye test in the three groups of mice **(F)** Fecal water content in the three groups of mice. ^∗^*P* < 0.05, ^∗∗^*P* < 0.01, and ^∗∗∗^*P* < 0.001. *n* = 5–10 mice for each group.

The data presented above have shown that oral administration of penicillin, either alone or in combination with streptomycin, may increase 5-HT levels availability with concomitant up-regulation of HCN2 expression in the cecum epithelium. We have also detected HCN2 protein levels in the colon mucosa of mice treated with the antibiotics in comparison with their respective controls. The results showed that co-administration of penicillin and streptomycin (P-S) or penicillin alone also led to significant up-regulation of HCN2 expression in the colonic mucosa, in addition to the cecum mucosa (Supplementary Figures 3A,B). Streptomycin-treated mice only showed small (statistically insignificant) increase of TPH1 expression in the colon mucosa (Supplementary Figure 3C). We then tested whether the increased HCN2 expression might be responsible for the elevated serum 5-HT and slower intestinal motility following exposure to penicillin. Therefore, mice (*n* = 5–10 for each group) were given daily intraperitoneal injection of Ivabradine (Iva, 20 mg/kg) while the mice were treated with penicillin. Another group of penicillin-treated mice were given saline as vehicle control. As shown in Figure 6, Iva treatment significantly attenuated the effects of penicillin on serum 5-HT level, cecum size and weight and fecal water content. Iva treatment also resulted in shorter intestinal transit time compared with the penicillin-treated vehicle control group, although the difference did not reach statistical significance. The results suggested that HCN2 channels may be involved in the intestinal flora-host interaction by regulating the secretion of 5-HT.

## Discussion

The primary aim of the current study was to test the hypothesis that the pacemaker channel HCN2, previously known to be expressed on GLP-1-secreting L cells, may play a role in the responses of enterochromaffin cells to altered intestinal flora due to oral administration of penicillin. For this purpose, conventional SPF mice were given penicillin G, one of the most frequently used antibiotics for Gram^+^ bacterial infections, in drinking water for a period of 10 days. For comparison, streptomycin, which is used for Gram^–^ bacterial infections, were administered to separate groups of mice, also in drinking water for 10 days. It was quite apparent that exposure of mice to the antibiotics was sufficient to cause disruptions of the intestinal flora compositions, with consequent changes in serum 5-HT level, cecum size and weight and intestinal functions. In support of our hypothesis, HCN2 immunofluorescence was detected on intestinal EC cells and penicillin treatment caused upregulation of HCN2 expression in cecum and colon mucosa. Streptomycin treatment, however, caused upregulation of TPH1, the rate-limiting enzyme for 5-HT metabolism. Our results suggest that antibiotics exposure may alter 5-HT availability and intestinal function through mechanisms that may vary depending on the antibacterial spectrum.

We found that the mice treated with antibiotics all had larger and heavier cecum than the control mice, which was consistent with the previous studies (Ge et al., 2017; De Vadder et al., 2018). Cecum is a storage site of intestinal flora and an important digestive organ in some species. Mammiferous cecum is inhabited with specific symbiotic bacteria, which could secrete cellulase to digest cellulose in food (Delpretti et al., 2013). Antibiotics intake may cause a significant decrease of the cellulose-degrading microorganisms. Accordingly, fibrous food may not be fully digested and may abnormally accumulate, leading to significant enlargement of the cecum. Cecum is also an important immune organ. Conceivably, bidirectional interaction between the flora and immune system may also be involved in the enlargement of cecum following antibiotic treatment. The exact mechanisms notwithstanding, cecum enlargement appears to be a typical manifestation of antibiotic-induced intestinal dysbiosis in mice.

Enteroendocrine cells are strategically positioned in the gut to sense changes in luminal contents including bacterial products. EC cells, by far the most abundant enteroendocrine cell type, synthesize and release 5-HT, a hormone with wide spread actions within and outside GIT. Previous studies have demonstrated functional HCN2 channels in some endocrine cells including the GLP-1-secreting L cells in the gut (Reimann et al., 2005; Zhang et al., 2009; Calejo et al., 2014). In the current study, we show that EC cells also express HCN2 immunoreactivity. Indeed, we found that within the gut, HCN2 immunoreactivity was most enriched in enteroendocrine cells and was co-labeled with 5-HT immunoreactivity, with weak expression also detected in the enteric nervous system. We speculate that HCN2 channel may play an important role in the regulation of EC cells excitability and 5-HT release.

Our observations showed that either penicillin or streptomycin treatment for 10 days was sufficient to significantly increase serum 5-HT levels and fecal water content in mice. 5-HT is known to promote intestinal motility and intestinal fluid secretion through interacting with 5-HT_3_ and 5-HT_4_ receptors (Kojima et al., 2017; Bhattarai et al., 2018; Stakenborg et al., 2019). Intriguingly, however, penicillin-treated mice showed significantly prolonged intestinal transit time, in spite of the increased 5-HT availability. The reason for the slowed intestinal transit was not clear but seemed to be independent of 5-HT signaling, since streptomycin-treated group had similarly elevated serum 5-HT level without showing prolongation of intestinal transit time. Previously, mice treated with a combination of four antibiotics (ampicillin, neomycin sulfate, metronidazole, and vancomycin) for 4 weeks displayed decreased intestinal motility with concomitant decrease of 5-HT availability, whereas administration of vancomycin alone for 7 days resulted in prolongation of intestinal transit time with concomitant increase of GLP-1 positive cells and serum GLP-1 levels (Ge et al., 2017; Xu et al., 2019). These data together suggest that intestinal dysbiosis due to antibiotics treatments may impact intestinal motility via different mechanisms depending on the antibacterial spectrum of the antibiotics administered.

Short-chain fatty acids (SCFAs) are the main metabolites of intestinal flora which may interact with epithelial cells including enteroendocrine cells (Larraufie et al., 2018; Lund et al., 2018). Acetic acid, propionic acid, and butyric acid estimatedly account for about 95% of total SCFAs in intestinal tract (Morrison and Preston, 2016; Sun et al., 2017). We found that both penicillin and streptomycin treatments for 10 days caused significant reductions in the content of SCFAs in cecum of mice, but the resultant cecum SCFAs profiles were obviously different between penicillin and streptomycin-treated mice. Accordingly, the penicillin-treated mice had more dramatic decreases in butyrate, isobutyrate and isovalerate levels than the streptomycin-treated mice, with the streptomycin group showing greater reduction in valerate. These results are consistent with the notion that penicillin and streptomycin treatments may alter the intestinal flora structure differently due to their different antibacterial spectrum. It was also noted that both penicillin and streptomycin treatments caused increases in propionic acid levels in the cecum. Although propionic acid is one of the main fermentation products of intestinal microorganisms with an anti-inflammatory role by modulating regulatory T (T_reg_) cells, excessive concentration of propionic acid may impair mitochondrial function (Frye et al., 2013, 2015; Furusawa et al., 2013; Murugesan et al., 2018). *Clostridium spp.* is the main intestinal bacteria to produce propionic acid (Macfabe, 2012, 2013). The increase of propionic acid in cecum indicated that either penicillin or streptomycin-induced disturbance of intestinal flora may favor the proliferation of *Clostridium spp.* Studies have indicated that elevated intestinal propionic acid content and *Clostridium spp.* density may be related to neuropsychiatric diseases, such as the autism spectrum disorders (Macfabe et al., 2007; Shuaib et al., 2012; Frye et al., 2013; Dejean de la Batie et al., 2014; Ding et al., 2017; Coretti et al., 2018; de la Batie et al., 2018).

Short chain fatty acids interact with the intestinal epithelium by activating specific G protein-coupled receptors. Studies have demonstrated that EC cells in small intestine and colon express FFAR3 and FFAR2 (Bellono et al., 2017; Lund et al., 2018). FFAR3 is coupled to G_q_ or G_i/o_ and FFAR2 specifically activates the G_i/o_ pathways (Brown et al., 2003; Le Poul et al., 2003; Nilsson et al., 2003). Activation of either FFAR2 or FFAR3 may down-regulate cAMP levels in EC cells. Conceivably, the decrease of cecal SCFAs contents may raise cAMP level in EC cells, which may in turn promote activation of HCN2 channel and 5-HT release.

We found that penicillin treatment caused significant upregulation of HCN2 expression in the cecal and colonic mucosa, indicating that HCN2 might be primarily responsible for the increased serum 5-HT level and the concomitant increases in fluid secretion (increased fecal water content). Indeed, Ivabradine (Iva), a blocker of HCN channel, not only attenuated the elevation of serum 5-HT and increase of fecal water content, but also ameliorated cecum enlargement associated with penicillin treatment. In contrast to penicillin, streptomycin treatment did not significantly alter HCN2 expression but resulted in an increase of TPH1 expression in the cecal epithelium, indicating that the elevation of serum 5-HT might be primarily due to increased 5-HT synthesis. However, it was previously reported that a combination of four antibiotics (ampicillin, neomycin sulfate, metronidazole, and vancomycin) for 4 weeks caused significant reduction of TPH1 mRNA expression and the number of 5-HT positive cells in colon of mice (Ge et al., 2017). Furthermore, those mice (treated with four antibiotics) displayed significantly less body weight gain than non-treated mice, whereas we found that mice treated with penicillin and streptomycin, either alone or in combination for 10 days, displayed similar body weight gain as the control mice. The reason for such discrepancies is not very clear but may be related to differences in the intestinal flora structure as a result of the different antibiotic treatments. Conceivably, the intestinal flora structure was altered much more robustly in the study of Ge et al. (2017) than in the current investigation. These data together suggest that antibiotics exposure may alter EC cells function and peripheral 5-HT availability through diverse mechanisms depending on the antibacterial spectrum. The current findings support the HCN2 channel playing a role in the communication between EC cells and the intestinal flora.

## Conclusion

The current study has shown that disruptions of the intestinal flora structure due to oral administration of penicillin may cause significant increases in serum 5-HT levels and changes in intestinal functions, at least partially mediated through HCN2 signaling. Oral administration of streptomycin may alter 5-HT availability by up-regulating TPH1 expression thus increasing synthesis of 5-HT. Alterations of the intestinal flora composition due to antibiotics treatment may alter peripheral 5-HT availability through different mechanisms, depending on the antibacterial spectrum.

## Data Availability Statement

The raw data supporting the conclusions of this article will be made available by the authors, without undue reservation.

## Ethics Statement

The animal study was reviewed and approved by the Ethnic Committee for Experimental Use of Animals of Shanghai Jiao Tong University School of Medicine.

## Author Contributions

WR, GZ, and XS conceived the study and obtained funding for these experiments. CZ and HG performed the experiments and analyzed the data. PL provided the technical support. CZ drafted the manuscript. WR and XS critically revised the manuscript. All authors have read and approved the final manuscript.

## Conflict of Interest

The authors declare that the research was conducted in the absence of any commercial or financial relationships that could be construed as a potential conflict of interest.

## Supplementary Material

Supplementary Figure 1 Oral administration of antibiotics did not significantly affect the body weight gain of mice. **(A)** Body weight gain in control mice and mice treated with penicillin and streptomycin (P-S). **(B)** Body weight gain in control mice and mice treated with penicillin alone. **(C)** Body weight gain in control mice and mice treated with streptomycin alone. **(D)** Body weight gain in mice treated with penicillin with or without Ivabradine (Iva) treatment. *n* = 5–10 mice for each group.

Supplementary Figure 2 Oral administration of penicillin did not significantly alter the EC cell density in mouse colon. **(A)** Representative microphotographs of 5-HT immunofluorescence (red) in the colon of control and penicillin-treated mice. **(B)** Bar graph showing that the average number of 5-HT positive cells in penicillin-treated mice was not significantly different from the control mice (each dot represents total number of 5-HT positive cells in 5 fields of the colonic tissue sections from one animal).

Supplementary Figure 3 HCN2 and TPH1 protein expression in the colon mucosa of control and antibiotics-treated mice. **(A)** Western blot detection of HCN2 and TPH1 expression in the colon mucosa of control mice and mice treated with penicillin + streptomycin (P-S); **(B)** HCN2 and TPH1 protein expression in the colon mucosa of control and penicillin- treated mice; **(C)** HCN2 and TPH1 protein expression in the colon mucosa of control and streptomycin-treated mice ^∗∗^*P* < 0.01, *n* = 5–6 mice for each group.
