# Supplementary figures and images for: Oral Administration of Penicillin or Streptomycin May Alter Serum Serotonin Level and Intestinal Motility via Different Mechanisms
Source: Front Physiol. 2020 Dec 23;11:605982. doi: 10.3389/fphys.2020.605982 (PMC7785965; doi:10.3389/fphys.2020.605982)

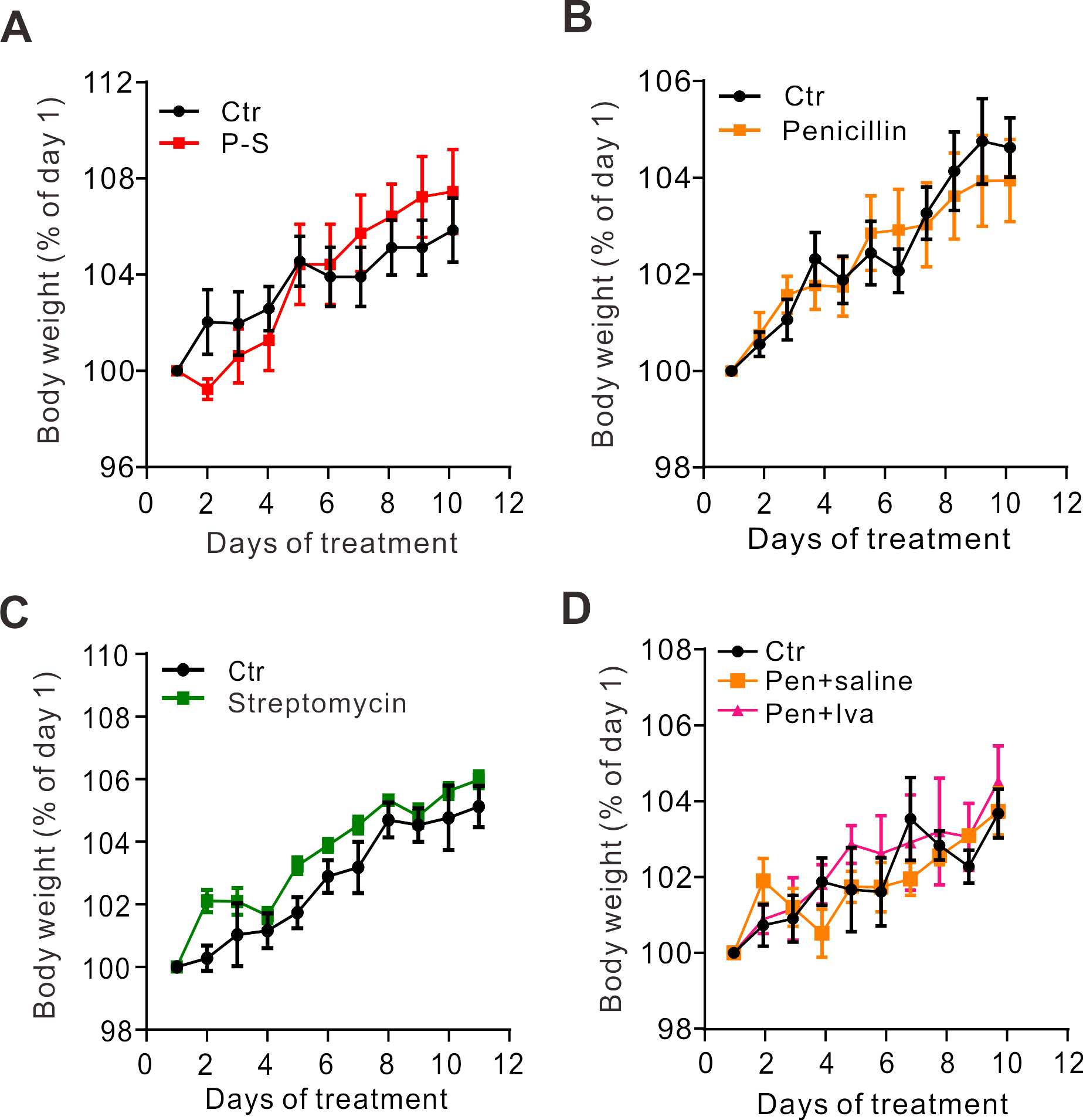

Supplement: Supplementary Figure 1 — Oral administration of antibiotics did not significantly affect the body weight gain of mice. (A) Body weight gain in control mice and mice treated with penicillin and streptomycin (P-S). (B) Body weight gain in control mice and mice treated with penicillin alone. (C) Body weight gain in control mice and mice treated with streptomycin alone. (D) Body weight gain in mice treated with penicillin with or without Ivabradine (Iva) treatment. n = 5–10 mice for each group. [file Image_1.TIF]

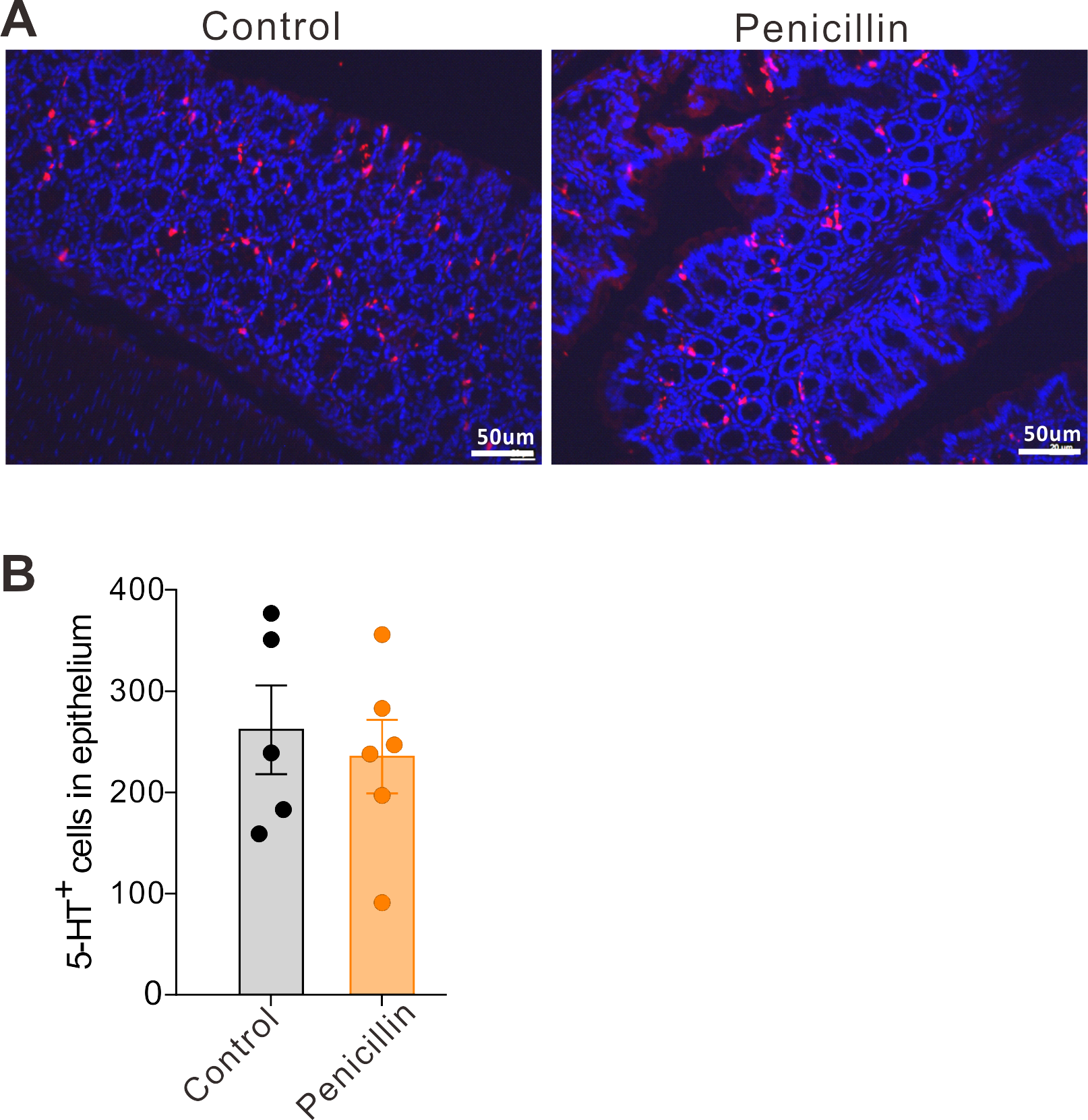

Supplement: Supplementary Figure 2 — Oral administration of penicillin did not significantly alter the EC cell density in mouse colon. (A) Representative microphotographs of 5-HT immunofluorescence (red) in the colon of control and penicillin-treated mice. (B) Bar graph showing that the average number of 5-HT positive cells in penicillin-treated mice was not significantly different from the control mice (each dot represents total number of 5-HT positive cells in 5 fields of the colonic tissue sections from one animal). [file Image_2.TIF]

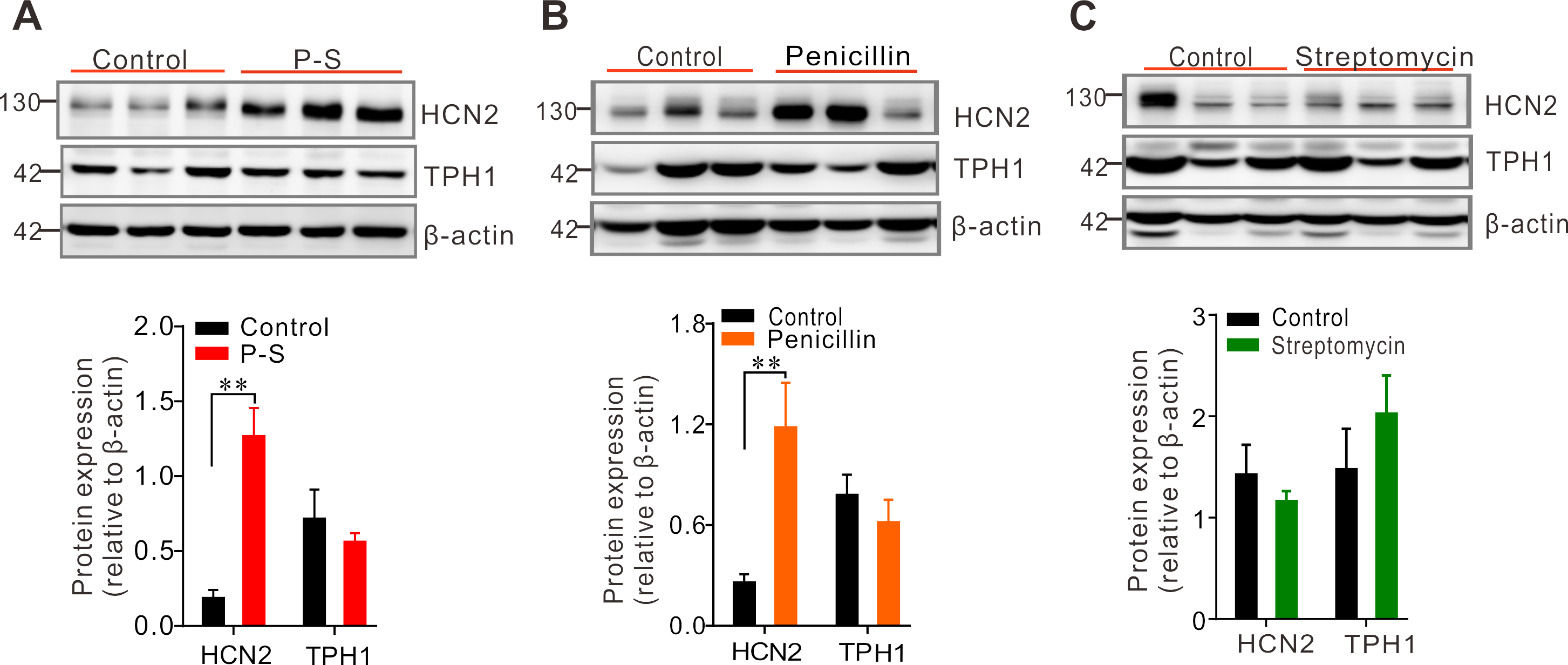

Supplement: Supplementary Figure 3 — HCN2 and TPH1 protein expression in the colon mucosa of control and antibiotics-treated mice. (A) Western blot detection of HCN2 and TPH1 expression in the colon mucosa of control mice and mice treated with penicillin + streptomycin (P-S); (B) HCN2 and TPH1 protein expression in the colon mucosa of control and penicillin- treated mice; (C) HCN2 and TPH1 protein expression in the colon mucosa of control and streptomycin-treated mice ∗∗P < 0.01, n = 5–6 mice for each group. [file Image_3.TIF]
